# Factors associated with the self-awareness of fall risk among older patients with osteoporosis: a cross-sectional analysis

**DOI:** 10.3389/fpubh.2026.1961139

**Published:** 2026-09-24

**Authors:** Hua Chen, Songmei Wu, Yujiao Lu, Xinyue Liang, Junxian Chen, Ziyang Jia, Xuezhi Liu, Erhuan Han

**Affiliations:** 1 School of Nursing and Health, Zhengzhou University, Zhengzhou, China; 2 Luoyang Orthopedic-Traumatological Hospital of Henan Province (Henan Provincial Orthopedic Hospital), Zhengzhou, China; 3 School of Nursing, Henan University of Chinese Medicine (Smart Health and Nursing Industry College), Zhengzhou, China

**Keywords:** older adults, falls, influencing factors, osteoporosis, self-awareness of fall risk

## Abstract

**Objectives:**

This study aimed to investigate the current status and influencing factors of self-awareness of fall risk among older patients with osteoporosis, and to provide a reference for developing evidence based fall prevention intervention strategies.

**Methods:**

A cross-sectional study was conducted. A convenience sampling method was employed to recruit older patients with osteoporosis from the osteoporosis departments of two campuses of a tertiary orthopaedic hospital in Henan Province from April to July 2026. Data were collected using a General Information Questionnaire, the Self-Awareness of Falls in Elderly Scale, the Barthel Index Scale, the Tilburg Frailty Indicator Scale, the Hospital Anxiety and Depression Scale and the Perceived Social Support Scale.

**Results:**

A total of 294 valid questionnaires were retrieved, yielding an effective response rate of 97.35%. The mean score of self-awareness of fall risk in older patients with osteoporosis was 64.53 ± 9.24. Multiple linear regression analysis revealed that age, gender, marital status, average monthly household income, living alone, fall prevention education, barthel index, depression and perceived social support were significant influencing factors (all *p* < 0.05), collectively explaining 42.0% of the total variance.

**Conclusion:**

Self-awareness of fall risk in older patients with osteoporosis varied across different dimensions and was influenced by a combination of multiple interrelated factors. Clinical nurses may implement targeted fall prevention interventions based on these factors to reduce the occurrence of fall related injuries.

## 1 Introduction

Osteoporosis (OP) is a systemic bone disease characterized by low bone mass and destruction of bone microarchitecture, leading to increased bone fragility and a significantly elevated risk of fractures (1). The global overall prevalence of osteoporosis is 23.1% in women and 11.7% in men (2); In China, the prevalence among older adults aged 60 years and above reaches as high as 37.7% (3). With the accelerating aging of the population, the prevalence of osteoporosis continues to rise (4), and it is projected that by 2050, the number of older patients with osteoporosis worldwide will exceed 500 million (5), imposing ever growing health burdens and socioeconomic costs.

The complications of osteoporosis include pain, spinal deformities and declines in physical function, among which falls and their subsequent fractures are the most severe consequences (6). Due to increased bone fragility, decreased skeletal muscle strength, bone loss, calcium and vitamin D deficiency, and activity restriction caused by fear of falling owing to pain, osteoporosis patients exhibit significantly lower muscle strength and balance capacity than populations without osteoporosis (7, 8). Falls have become the sixth leading cause of death among older adults aged 65 and above. A domestic study (9) reported that the fall incidence and recurrent fall rate among Chinese osteoporosis patients were 14.6 and 2.1%, respectively, with a postfall fracture rate as high as 29.9%. The most serious consequence of osteoporotic fractures is hip fracture, of which 95% are caused by falls (10). Due to its high mortality and disability rates, hip fracture has drawn considerable attention: within 1 year after the fracture, the disability rate among older adults is approximately 50%, and the mortality rate ranges from 20 to 30% (11, 12). Osteoporosis and falls play a decisive role in the causative factors of hip fractures in older adults, and falls have a tremendous impact on patients’ physical and mental health. Therefore, preventing falls is a critical step in ensuring patient safety and reducing injury occurrence.

The 2022 World Guidelines for Falls Prevention and Management in Older Adults (13) propose that the core to enhancing the effectiveness of fall management lies in clarifying the patient’ s role as the primary responsible party and their central position in prevention. Therefore, reducing the risk and incidence of falls in older patients with osteoporosis requires not only environmental modifications and exercise interventions, but also stimulating patients’ active participation and their alertness to fall risks. Existing studies have mostly focused on objective risk factors such as age and muscle strength, whereas the key subjective cognitive factor, self-awareness of fall risk, has been insufficiently explored. Self-awareness of fall risk is defined as older adults’ subjective perception of existing or potential fall risks based on their situational context, as well as their understanding of how various risk factors may contribute to falls (14). An in depth understanding of its formation mechanisms and pathways of action can help guide patients to accurately identify risks and adopt adaptive behaviors, thereby reducing falls.

Research on self-awareness of fall risk started earlier abroad, with studies on influencing factors and intervention trials conducted among hospitalized patients (15, 16) and community dwelling older adults (17), and the methodological framework is relatively mature. In China, however, this concept has only been introduced in recent years and remains in an exploratory stage overall. Existing domestic studies have largely focused on chronic disease populations such as cardiovascular disease (18, 19) and stroke (20, 21). To date, only one best evidence summary has addressed self-awareness of fall risk in the osteoporotic population, specifically among older postoperative patients with osteoporotic vertebral compression fracture (OVCF) (22). This summary itself explicitly acknowledged that “high-quality direct evidence in this research field remains limited” and called for further rigorous primary studies. Notably, that evidence summary is prescriptive in nature, providing recommendations on how to improve awareness, and its focus on a narrow postoperative OVCF subgroup leaves the broader older adults with osteoporosis largely unexamined. Most critically, no original quantitative study has systematically investigated the status and associated factors of self-awareness of fall risk in older adults with osteoporosis using a validated instrument.

Addressing this gap is clinically and publicly relevant. Given the unique disease characteristics of osteoporosis, including bone fragility, chronic pain and specific medication regimens, and the severe consequences of falls in this population, understanding their self-awareness of fall risk levels and associated factors is a crucial prerequisite for developing targeted fall prevention strategies. Therefore, this study aims to investigate the current status and influencing factors of self-awareness of fall risk in older patients with osteoporosis through a quantitative survey. At the clinical level, this study offers evidence to help identify osteoporosis patients with low self-awareness of fall risk enabling targeted screening and tailored interventions. At the public health level, falls in this population incur substantial economic burdens. Enhancing self-awareness of fall risk offers an affordable and widely applicable means of reducing fractures and improving patients’ quality of life.

## 2 Methods

### 2.1 Study design and participants

This study used a crosss-sectional survey research design. Using a convenience sampling method, older patients with osteoporosis who were hospitalized in the Department of Osteoporosis at two campuses of a tertiary orthopedic hospital in Henan Province from April to July 2026 were enrolled as study subjects. Inclusion criteria: ① age≥60 years; ② diagnosis of osteoporosis according to the Guidelines for the Diagnosis and Treatment of Primary Osteoporosis in China (2022) (23); ③ ability to correctly answer questions and complete the questionnaire independently or with assistance; ④ voluntary participation with signed informed consent. Exclusion criteria: ① severe neurological deficits or cognitive impairment; ② severe physical dysfunction, critical illness, or serious complications that prevented cooperation with the survey. Questionnaires were eliminated if: ① > 5% of items were incorrectly filled or missing; ② responses showed obvious regularity or identical answers throughout. The sample size was estimated according to Kendall’ s rule of thumb (24), which recommends 5 to 10 times the number of independent variables. After dummy coding of unordered categorical variables, a total of 25 candidate variables were included in this study. Accounting for a 20% attrition rate, the final sample size was determined to range from 156 to 313 participants. Additionally, an *a priori* power analysis was performed using G*Power software. Based on a medium effect size (*f*^2^ = 0.15), α = 0.05, power = 0.80, and 25 predictors, the estimated minimum sample size was 172 participants; after accounting for a 20% attrition rate, the target sample size was set at 215 participants. A total of 294 participants were ultimately enrolled, which fell within this range and met the sample size requirement of this study. Ethical approval was obtained from the Ethics Committee of Luoyang Orthopedic-Traumatological Hospital of Henan Province (Henan Provincial Orthopedic Hospital), with approval number 2026YJSKT0024-02.

### 2.2 Measures

#### 2.2.1 General information questionnaire

The questionnaire was self-designed by the researchers based on literature review and group brainstorming. The demographic data included age, gender, educational level, marital status, average monthly household income, medical payment methods and living alone. Disease related data included history of falls in the past year, type of fall injuries, number of comorbid chronic diseases, use of walking aids, visual impairment, pain level, fall risk, self perceived sleep quality and fall prevention education.

#### 2.2.2 Self-awareness of falls in older adults scale (SAFE)

This scale was developed by Shyu et al. (14) in 2018 to measure self-awareness of fall risk in hospitalized older patients. In mainland China, He et al. (25) tested its reliability and validity among hospitalized older adults and reported a Cronbach’s α coefficient of 0.923. The SAFE scale comprises 21 items across four dimensions: (1) awareness of activity safety and environment: evaluating whether older patients are aware of the aspects of their daily activities and surrounding environment that could cause falls; (2) awareness of physical functions: evaluating whether older patients are aware of the aspects of their physical condition that could cause falls; (3) awareness of medication: assessing whether older patients are aware of the risk of falling caused by the medications they take; (4) awareness of cognitive behavior: examining the cognitive behaviors of older adult inpatients for fall prevention. The total SAFE score, calculated as the sum of all 21 items, reflects the individual’s overall level of self-awareness of fall risk, while subscale scores can be used to identify dimension-specific deficiencies. All items are rated on a 5-point Likert scale, with scores ranging from 1 (strongly agree) to 5 (strongly disagree). Items 1, 4, 6, 7, 8 and 15 are reverse-scored. Total scores range from 21 to 105, with higher scores indicating a higher level of self-awareness of fall risk.

#### 2.2.3 Barthel index scale(BI)

This scale was originally developed by Mahoney et al. (26) in 1965 and was translated into Chinese by Hou et al. (27) in 2012, with a Cronbach’s α coefficient of 0.916. It is used to assess activities of daily living (ADL). The scale includes 10 items: feeding, bathing, grooming, dressing, bowel control, bladder control, toilet use, bed-to-chair transfer, ambulation on level ground, and stair climbing. Total scores range from 0 to 100, with higher scores indicating better independence. Scores ≤40 indicate severe dependence, 41–60 moderate dependence, 61–99 mild dependence, and 100 complete independence.

#### 2.2.4 Tilburg frailty Indicator scale (TFI)

This scale was developed by Gobbens et al. (28) and was translated into Chinese by the Xi et al. (29) in 2013. It has a Cronbach’s α coefficient of 0.730. The instrument comprises 15 items across three domains: physical frailty, psychological frailty, and social frailty. Total scores range from 0 to 15. A total score≥5 indicates frailty, with higher scores reflecting greater severity of frailty.

#### 2.2.5 Hospital anxiety and depression scale(HADS)

This scale was developed by Zigmond and Snaith (30) to assess anxiety and depression in hospitalized patients. It consists of 14 items, with 7 odd-numbered items assessing anxiety and 7 even-numbered items assessing depression. Each item is scored on a 4-point Likert scale (0–3), and the total score for each subscale ranges from 0 to 21. Scores of 0–7 indicate no symptoms, 8–10 suggest possible mild emotional disturbance, and 11–21 indicate clinically significant anxiety or depressive symptoms. Higher total scores reflect greater negative affect. The Chinese version has a Cronbach’s α coefficient of 0.879 (31), demonstrating good reliability and validity.

#### 2.2.6 Perceived social support scale(PSSS)

This scale was developed by Zimet et al. (32) in 1990 and translated and revised by the Jiang et al. (33), with a Cronbach’s α coefficient of 0.92. It measures the degree of perceived support from family, friends, and others. The scale includes 12 items across three dimensions: family support, friend support, and other support. Higher scores indicate greater perceived social support. Items are rated on a 7-point Likert scale (1 = strongly disagree, 7 = strongly agree). Total scores range from 12 to 84, with 12–36 indicating low support, 37–60 moderate support, and 61–84 high support.

### 2.3 Data collection

After securing institutional support from department directors and head nurses, three trained investigators administered printed questionnaires to older patients with osteoporosis. Interviews were conducted individually using standardized instructions to explain the study objectives, questionnaire content, and completion procedures. Written informed consent was obtained prior to questionnaire distribution. Patients were encouraged to complete the questionnaires independently; when this was not feasible due to physical limitations, the investigators read the items aloud and recorded the patients’ responses accordingly. Completed questionnaires were immediately collected and reviewed on site for missing or erroneous entries. Any inconsistencies were resolved through verification with the participants to ensure data integrity.

### 2.4 Statistical analysis

Data were entered using Microsoft Excel and analyzed with SPSS version 27.0. A two-sided significance level was set atα = 0.05, and *p* < 0.05 was considered statistically significant. Categorical variables were described as frequencies and percentages. Continuous variables were expressed as mean±standard deviations (SD). Pearson correlation analysis was conducted to examine the relationships between the self-awareness of fall risk and barthel index, frailty, anxiety/depression and perceived social support. For univariate analyses, independent samples *t*-tests were used to compare means between two groups and one-way ANOVA was used for comparisons among three or more groups. Finally, candidate variables were first screened using univariate and correlation analyses, and those reaching statistical significance (*p* < 0.05) were then included in a multiple linear regression model, with the total SAFE score as the dependent variable.

## 3 Results

### 3.1 General information and self-awareness of fall risk status in older patients with osteoporosis

A total of 302 questionnaires were distributed, of which 294 were considered valid, yielding a valid response rate of 97.35%. The age of older patients with osteoporosis ranged from 60 to 91 years, with a mean of 68.94 (SD = 7.01). Detailed demographic characteristics and comparisons of self-awareness of fall risk scores are presented in Table 1. The overall self-awareness of fall risk score of the patients was 64.53 ± 9.24. The dimension specific scores for self -awareness of fall risk are shown in Table 2.

**Table 1:** Group comparison of self-awareness of fall awareness among older patients with osteoporosis (*N* = 294).

Characteristics	*n* (%)	Mean±SD	*F/t*	*p*
Age				
60~	169 (57.5%)	62.55 ± 8.62	21.513*	<0.001
70~	95 (32.3%)	65.15 ± 8.14		
80~	30 (10.2%)	73.70 ± 10.28		
Gender				
Male	28 (9.5%)	59.93 ± 8.71	−2.801**	0.005
Female	266 (90.5%)	65.01 ± 9.18		
Education level				
Primary school or below	84 (28.6%)	63.49 ± 9.29	1.209*	0.307
Junior high school	75 (25.5%)	64.95 ± 9.88		
High school/technical secondary school	84 (28.6%)	65.85 ± 8.90		
College degree or above	51 (17.3%)	63.45 ± 8.64		
Marital status				
Married	257 (87.4%)	63.57 ± 8.56	−4.033**	<0.001
Unmarried/Divorced/Widowed	37 (12.6%)	71.19 ± 11.03		
Average monthly household income(RMB)				
<3,000	103 (35.0%)	62.27 ± 8.73	4.895*	0.008
3,000–5000	150 (51.0%)	65.64 ± 8.92		
>5,000	41 (13.9%)	66.12 ± 10.69		
Medical payment methods				
Urban and rural residents’ medical insurance	137 (46.6%)	62.34 ± 8.08	7.590*	0.001
Employee medical insurance	139 (47.3%)	66.51 ± 9.88		
Self pay	18 (6.1%)	65.83 ± 9.17		
Living alone				
Yes	10 (3.4%)	70.30 ± 9.91	2.021**	0.044
No	284 (96.6%)	64.32 ± 9.17		
History of falls in the past year				
None	150 (51.0%)	62.05 ± 7.72	12.380*	<0.001
1–2 times	132 (44.9%)	66.79 ± 9.96		
3–4 times	12 (4.1%)	70.67 ± 9.93		
Type of fall injuries				
None	147 (50.0%)	62.04 ± 7.94	6.368*	<0.001
Bruise	19 (6.5%)	64.89 ± 6.64		
Abrasion	33 (11.2%)	63.88 ± 8.13		
Bleeding	11 (3.7%)	68.55 ± 10.89		
Fracture	84 (28.6%)	68.52 ± 10.57		
Number of comorbid chronic diseases				
None	99 (33.7%)	62.65 ± 8.47	3.888*	0.022
1	119 (40.5%)	64.75 ± 8.71		
≥2	76 (25.9%)	66.63 ± 10.54		
Use of walking aids				
Yes	45 (15.3%)	69.29 ± 9.15	3.844**	<0.001
No	249 (84.7%)	63.67 ± 9.01		
Visual impairment				
Yes	177 (60.2%)	65.06 ± 9.49	1.222**	0.223
No	117 (39.8%)	63.72 ± 8.83		
Pain level				
No pain	17 (5.8%)	66.65 ± 9.29	2.900*	0.057
Mild pain	237 (80.6%)	63.90 ± 9.08		
Moderate pain	40 (13.6%)	67.35 ± 9.73		
Falls risk				
Low	74 (25.2%)	60.97 ± 7.59	12.820*	<0.001
Moderate	124 (42.2%)	64.14 ± 8.80		
High	96 (32.7%)	67.77 ± 9.90		
Self perceived sleep quality				
Good	51 (17.3%)	62.24 ± 9.63	2.754*	0.065
Fair	161 (54.8%)	64.47 ± 8.56		
Poor	82 (27.9%)	66.07 ± 10.05		
Fall prevention education				
Never	147 (50.0%)	62.90 ± 8.08	8.062*	<0.001
Occasionally	127 (43.2%)	65.29 ± 9.81		
Frequently	20 (6.8%)	71.60 ± 9.99		

^*^F, ^**^t.

**Table 2:** Total and dimension scores of self-awareness of fall risk in older patients with osteoporosis (*N* = 294).

Item	Minimum	Maximum	Total score (Mean ± SD)	Average scores (Mean ± SD)
Self-awareness of fall risk	46	88	64.53 ± 9.24	3.07 ± 0.44
Awareness of activity safety and environment	22	38	29.48 ± 3.45	3.69 ± 0.43
Awareness of physical functions	9	27	17.14 ± 3.94	2.86 ± 0.66
Awareness of medication	4	14	7.77 ± 2.07	2.59 ± 0.69
Awareness of cognitive behavior	5	17	10.14 ± 2.71	2.53 ± 0.68

### 3.2 Univariate analysis of factors associated with self-awareness of fall risk in older patients with osteoporosis

Univariate analysis revealed that age, gender, marital status, average monthly household income, medical payment methods, living alone, history of falls in the past year, type of fall injuries, number of comorbid chronic diseases, use of walking aids, fall risk and fall prevention education were significantly associated with self-awareness of fall risk scores in older patients with osteoporosis (all *p* < 0.05). Detailed results are presented in Table 1.

### 3.3 Correlations between self-awareness of fall risk and Barthel index, frailty, anxiety, depression and perceived social support

Correlation analysis showed that the barthel index score of older patients with osteoporosis ranged from 30 to 100, with a mean of 87.76 ± 12.97, and was negatively correlated with SAFE. The total frailty score ranged from 0 to 13, with a mean of 5.11 ± 2.66, and was positively correlated with SAFE. The total anxiety score ranged from 0 to 13, with a mean of 4.63 ± 2.48, and was positively correlated with SAFE. The total depression score ranged from 2 to 18, with a mean of 7.78 ± 3.00, and was positively correlated with SAFE. The perceived social support score ranged from 44 to 73, with a mean of 60.58 ± 5.46, and was also positively correlated with SAFE. Detailed results are displayed in Table 3.

**Table 3:** Result correlation analysis between study variables and self-awareness of fall risk (*N* = 294).

Variable	Self-awareness of fall risk	Barthel index	Fragility	Anxiety	Depression	Perceived social support
Self-awareness of fall risk	1					
Barthel index	−0.292**	1				
Fragility	0.297**	−0.488**	1			
Anxiety	0.121*	−0.291**	0.592**	1		
Depression	0.210**	−0.340**	0.679**	0.690**	1	
Perceived social support	0.286**	0.161**	−0.256**	−0.229**	−0.339	1

*p < 0.05, **p < 0.01.

### 3.4 Multiple linear regression analysis of self-awareness of fall risk in older patients with osteoporosis

Using the self-awareness of fall risk score as the dependent variable, and the statistically significant variables from univariate and correlation analyses as independent variables, a multiple linear regression analysis was performed after variable assignment, with the specific assignments detailed in Table 4. The results indicated that age, gender, marital status, average monthly household income, living alone, fall prevention education, barthel index, depression and perceived social support were independent influencing factors of self-awareness of fall risk in older patients with osteoporosis (*p* < 0.05), collectively explaining 42.0% of the total variance, as shown in Table 5.

**Table 4:** Independent variable assignment.

Independent variable	Assignment method
Age	1 = 60~, 2 = 70~, 3 = 80~
Gender	0 = Male, 1 = Female
Marital status	0 = Married, 1 = Unmarried/Divorced/Widowed
Average monthly household income(RMB)	1 = <3,000, 2 = 3,000 ~ 5,000, 3 = >5,000
Medical payment methods	Dummy variables were set using “Self pay” as the control group: Self pay (*Z*_1_ = 0, *Z*_2_ = 0), Urban and rural residents’ medical insurance (*Z*_1_ = 1, *Z*_2_ = 0), Employee medical insurance (*Z*_1_ = 0, *Z*_2_ = 1)
Living alone	0 = No, 1 = Yes
History of falls in the past year	1 = None, 2 = 1–2times, 3 = 3–4 times
Type of fall injuries	Dummy variables were set using“None”as the control group: None (*Z*_1_ = 0, *Z*_2_ = 0, *Z*_3_ = 0, *Z*_4_ = 0), Bruise (*Z*_1_ = 1, *Z*_2_ = 0, *Z*_3_ = 0, *Z*_4_ = 0), Abrasion (*Z*_1_ = 0, *Z*_2_ = 1, *Z*_3_ = 0, *Z*_4_ = 0), Bleeding (*Z*_1_ = 0, *Z*_2_ = 0, *Z*_3_ = 1, *Z*_4_ = 0); Fracture (*Z*_1_ = 0, *Z*_2_ = 0, *Z*_3_ = 0, *Z*_4_ = 1)
Number of comorbid chronic diseases	1 = None, 2 = 1, 3 = ≥2
Use of walking aids	0 = No, 1 = Yes
Falls risk	1 = Low, 2 = Moderate, 3 = High
Fall prevention education	1 = Never, 2 = Occasionally, 3 = Frequently

**Table 5:** Results of multiple linear regression analysis of self-awareness of fall risk in older patients with osteoporosis (*N* = 294).

Variable	Regression coefficient	Standard error	Standardized regression coefficient	*t*	*p*
Constant	24.263	7.570	—	3.205	0.002
Age	1.870	0.772	0.137	2.422	0.016
Gender	4.545	1.463	0.145	3.107	0.002
Married status	3.032	1.411	0.109	2.149	0.033
Average monthly household income (RMB)	1.999	0.938	0.145	2.132	0.034
Living alone	5.468	2.416	0.107	2.263	0.024
Fall prevention education	2.278	0.824	0.153	2.765	0.006
Barthel index	−0.150	0.043	−0.210	−3.446	<0.001
Depression	0.764	0.228	0.248	3.350	<0.001
Perceived social support	0.539	0.090	0.318	5.967	<0.001

*R* = 0.680, *R*^2^ = 0.462, adjusted *R*^2^ = 0.420, *F* = 11.121, *p* < 0.001; The full model (including all predictors, VIF and Tolerance) is available in Supplementary Table S1.

Partial regression plots confirmed a linear relationship between the independent variables and fall vigilance scores. The scatterplot of standardized residuals against standardized predicted values showed a random distribution, suggesting homoscedasticity. Histograms and P–P plots indicated an approximately normal distribution of standardized residuals. The Durbin-Watson statistic was 1.838, indicating independence of residuals. For all independent variables, the VIF values ranged from 1.096 to 5.497, and the Tolerance values ranged from 0.182 to 0.913, all of which were within the recommended limits (VIF < 10, Tolerance>0.1), indicating no serious multicollinearity (34). These results confirm that all assumptions of the linear regression model were adequately met, supporting the reliability of the findings.

## 4 Discussion

The mean SAFE score in our osteoporosis sample was higher than that reported in general older inpatients (35). This may be because osteoporosis patients have a good understanding of the increased fall risk and severe consequences due to increased bone fragility, and some patients have experienced fall related trauma. In addition, these patients often require long term antiosteoporosis medication and regular follow up visits, which provide them with more opportunities to obtain fall prevention health information from healthcare professionals, thus contributing to their higher alertness. Among the subscale scores, the highest score was observed in the dimension of awareness of activity safety and environment, a finding that is consistent with the study by Cui et al. (36). This may be attributed to the fact that osteoporosis, as an age related bone disease, can lead to fractures even under minor external force, which renders patients particularly sensitive to environmental hazards such as wet floors and insufficient lighting. By actively identifying potential safety risks in their daily activities and living environments, patients’ awareness of fall risk is significantly enhanced. In contrast, the lowest score was recorded in the dimension of awareness of cognitive behavior, which aligns with the results reported by Deng et al. (18). This discrepancy may be explained by the following considerations. In our sample, patients aged 60–70 years accounted for 57.5% of the total; this age group still retains a certain level of self care ability and may therefore pay insufficient attention to their own health. Moreover, falls are often attributed by some patients to accidental events, and the risk of fractures is frequently underestimated. Furthermore, half of the participants (50.0%) had never received formal fall prevention education, resulting in a lack of systematic knowledge essential for developing proactive risk avoidance behaviors. In addition, a “refusing to admit aging” mentality exists among some patients, which leads to poor adherence to fall prevention education and further weakens their alertness at the cognitive behavioral level.

In this study, age was identified as an influencing factor of self-awareness of fall risk among older patients with osteoporosis, a finding that is consistent with the report by Xu et al. (37). This relationship may be attributable to the age related decline in physical function, which intensifies patients’ subjective perception of fall risk. Moreover, older patients often have a history of negative health events which provides them with a clear awareness of the severe outcomes of falls, thus sustaining a high level of vigilance (38). Furthermore, male patients demonstrated lower self-awareness of fall risk than female patients, in agreement with the study by Xia et al. (39). This difference may be explained by women’s generally greater sensitivity to health threats and their stronger initiative in health information seeking. In addition, the higher prevalence of osteoporosis among older women, along with the longer rehabilitation periods and more frequent complications following fractures, further reinforces their cautious behavior in daily life and elevates their alertness (40). Our results also showed that patients with higher average monthly family income tended to have higher self-awareness of fall risk, which is consistent with Li et al. (41). This may be due to the fact that higher income groups possess better access to health resources and greater medical adherence, enabling them to actively acquire osteoporosis related protective knowledge through diverse information channels and develop a comprehensive awareness of potential risks, thereby achieving a more thorough perception of fall risk (42). These findings highlight the necessity for healthcare professionals to develop stratified intervention strategies tailored to individual patient characteristics. For older and female patients who exhibit heightened alertness, close monitoring is warranted to prevent the vicious cycle in which fear of falling precipitates kinesiophobia and subsequent restriction of physical activity. For male and low income patients, visual risk communication using fracture risk assessment tools should be adopted to overcome cognitive barriers, thereby effectively enhancing their self-awareness of fall risk and promoting active prevention behaviors.

In this study, married older patients with osteoporosis had lower self-awareness of fall risk scores than those who were unmarried, divorced, or widowed, which is consistent with the findings of Zhou et al. (43). This may be explained by the fact that married patients are more likely to receive fall related risk information and daily behavioral supervision from their spouses, which directly enhances their risk perception and promotes psychological well-being, thereby influencing their level of self-awareness of fall risk (44). In addition, living alone was identified as an influencing factor of self-awareness of fall risk in older patients with osteoporosis. This may be attributed to the higher fall rate among older adults living alone and the lack of timely assistance and care in the event of a fall (45). Such circumstances raise concerns about delayed medical treatment or even life threatening consequences, which compel these patients to maintain a high level of vigilance in daily life (46). Furthermore, patients who had received fall prevention education more frequently in the past demonstrated suggests that systematic health education can effectively improve patients’ awareness of fall risk and motivate them to actively engage in fall prevention (47). Meanwhile, older adults with dependent self care ability exhibited higher self-awareness of fall risk, in agreement with Liu et al. (48). This may be related to their clear awareness of their limited mobility and the potential consequences of falls, which reduces their confidence in performing daily activities and thus sustains a higher alert level (49). In contrast, those with better self care ability tended to be more optimistic about their physical capacity and underestimated fall risk, resulting in relatively lower alertness. Therefore, for older patients with osteoporosis who lack spousal support or live alone, healthcare professionals should establish a community family integrated regular follow up mechanism and actively increase the frequency of telephone follow ups. At the same time, diversified fall prevention education strategies, such as case based warnings and scenario simulations, should be adopted to correct patients’ cognitive biases regarding fall risk, thereby comprehensively improving their self-awareness of fall risk.

Our study found that older patients with osteoporosis with depressive symptoms had higher self-awareness of fall risk, which is consistent with the findings of Li et al. (50). A higher self-awareness of fall risk does not necessarily indicate better psychological health or more effective fall-prevention behavior. Instead, this positive association may reflect heightened threat perception or psychological distress frequently accompanied by depressive symptoms. Depressed patients may exhibit a state of hypervigilance toward potential dangers, leading to increased self-awareness of fall risk. In addition, our results showed that older patients with osteoporosis who had higher levels of social support had better self-awareness of fall risk, in agreement with the report by Jin et al. (51). Patients with abundant social support tend to have active social networks and diverse channels for health information acquisition, and they more frequently receive health risk reminders and positive guidance from family members, communities or healthcare professionals. This repetitive input of safety information may increase their cognitive accessibility to potential hazards, thereby reinforcing their attention to fall risks (52). Additionally, perceived emotional support may act as a buffer against psychosocial stress, enabling individuals to process risk information more calmly and rationally. However, these specific psychological mechanisms were not directly tested, and our interpretation remains speculative. Therefore, healthcare professionals should attend to patients’ negative emotions in clinical practice and provide timely psychological counseling. At the same time, efforts should be made to engage family caregivers, patient peer support groups and community resources to establish a diversified social support system. Regular peer education and exchange activities should be organized to improve patients’ self-awareness of fall risk and reduce the occurrence of fall events.

## 5 Conclusion

Self-awareness of fall risk in older patients with osteoporosis was influenced by multiple factors, including age, gender, marital status, average monthly family income, living alone, previous fall-prevention education, Barthel index, depression, and social support. In clinical practice, when assessing self-awareness of fall risk in this population, healthcare professionals should pay close attention to the comprehensive interaction of these multiple factors, particularly considering patient’ functional status, psychological well-being and available social support. It is also suggested that future longitudinal or interventional studies focus on patients’ psychosocial status, reinforce systematic fall prevention education, and integrate family and community resources to test whether building a strong social support network can effectively enhance self-awareness of fall risk and self management abilities. Ultimately, prospective trials are required to determine whether these strategies reduce the incidence of falls and fractures and improve patients’ quality of life.

## 6 Limitations

We acknowledge several limitations in the present study. First, the cross-sectional design precludes causal inference, and the use of convenience sampling within a single hospital limits the generalizability of our findings. Second, the reliance on self-reported data may introduce recall bias, while face-to-face interviews may introduce interviewer bias. Third, the highly imbalanced sex distribution limits the applicability of our conclusions to male patients. Finally, the lack of longitudinal fall outcomes and the absence of direct assessment regarding whether higher awareness translates into fall-prevention behaviors remain unresolved. Therefore, future research should adopt multicenter, longitudinal prospective cohort designs, incorporate objective follow-up data such as electronic medical records, and test mediating mechanisms to clarify the dynamic impact and causal pathways of heightened awareness on actual fall prevention behaviors. Moreover, the observed associations require the use of more objective and comprehensive exposure assessment methods in the future and verification through prospective cohort studies to validate.

## Data Availability

The original contributions presented in the study are included in the article/Supplementary material, further inquiries can be directed to the corresponding author.
