# Supplementary material for: Factors associated with the self-awareness of fall risk among older patients with osteoporosis: a cross-sectional analysis
Source: Front Public Health. 2026 Sep 24;14:1961139. doi: 10.3389/fpubh.2026.1961139 (PMC13645558; doi:10.3389/fpubh.2026.1961139)
Supplement: Supplementary file 1 [file Table_1.docx]

**Supplementary Table S1**

| **Variable** | | **Regression coefficient** | **Standard error** | **Standardized regression coefficient** | ***t*** | ***P*** | **Tolerance** | **VIF** |
| --- | --- | --- | --- | --- | --- | --- | --- | --- |
| Constant | | 24.263 | 7.570 | — | 3.205 | 0.002 | — | — |
| Age | | 1.870 | 0.772 | 0.137 | 2.422 | 0.016 | 0.622 | 1.607 |
| Gender | | 4.545 | 1.463 | 0.145 | 3.107 | 0.002 | 0.913 | 1.096 |
| Married status | | 3.032 | 1.411 | 0.109 | 2.149 | 0.033 | 0.768 | 1.302 |
| Average monthly household income(RMB) | | 1.999 | 0.938 | 0.145 | 2.132 | 0.034 | 0.430 | 2.327 |
| Medical payment methods  “Self pay” as the control group | Urban and rural residents’ medical insurance | -3.551 | 1.889 | -0.192 | -1.880 | 0.061 | 0.190 | 5.276 |
|  | Employee medical insurance | -2.936 | 1.916 | -0.159 | -1.533 | 0.127 | 0.184 | 5.439 |
| Living alone | | 5.468 | 2.416 | 0.107 | 2.263 | 0.024 | 0.877 | 1.140 |
| History of falls in the past year | | 2.051 | 1.630 | 0.128 | 1.259 | 0.209 | 0.192 | 5.222 |

| **Variable** | | **Regression coefficient** | **Standard error** | **Standardized regression coefficient** | ***t*** | ***P*** | **Tolerance** | **VIF** |
| --- | --- | --- | --- | --- | --- | --- | --- | --- |
| Type of fall injuries  “None” as the control group | Bruise | 0.229 | 2.457 | 0.006 | 0.093 | 0.926 | 0.461 | 2.169 |
|  | Abrasion | 0.221 | 2.198 | 0.008 | 0.101 | 0.920 | 0.350 | 2.861 |
|  | Bleeding | 2.330 | 3.087 | 0.048 | 0.755 | 0.451 | 0.490 | 2.039 |
|  | Fracture | 2.303 | 2.129 | 0.113 | 1.082 | 0.280 | 0.182 | 5.497 |
| Number of comobid chronic diseases | | 0.016 | 0.699 | 0.001 | 0.023 | 0.982 | 0.584 | 1.713 |
| Use of walking aids | | -1.057 | 1.580 | -0.041 | -0.669 | 0.504 | 0.520 | 1.924 |
| Fall risk | | -0.368 | 1.028 | -0.030 | -0.358 | 0.721 | 0.278 | 3.600 |
| Fall prevention education | | 2.278 | 0.824 | 0.153 | 2.765 | 0.006 | 0.650 | 1.539 |
| Barthel Index | | -0.150 | 0.043 | -0.210 | -3.446 | ＜0.001 | 0.531 | 1.885 |
| Fragility | | 0.440 | 0.269 | 0.126 | 1.633 | 0.104 | 0.330 | 3.032 |
| Anxiety | | -0.446 | 0.244 | -0.120 | -1.831 | 0.068 | 0.463 | 2.159 |
| Depression | | 0.764 | 0.228 | 0.248 | 3.350 | ＜0.001 | 0.361 | 2.770 |
| Perceived social support | | 0.539 | 0.090 | 0.318 | 5.967 | ＜0.001 | 0.695 | 1.439 |

*R*=0.680, *R^2^*=0.462, adjusted *R^2^*=0.420, *F*= 11.121, *P*＜0.001;
